# Wave-Processing of Long-Scale Information by Neuronal Chains

**DOI:** 10.1371/journal.pone.0057440

**Published:** 2013-02-27

**Authors:** José Antonio Villacorta-Atienza, Valeri A. Makarov

**Affiliations:** Deptartment of Applied Mathematics, Facultad de Ciencias Matemáticas, Universidad Complutense, Madrid, Spain; Humboldt University, Germany

## Abstract

Investigation of mechanisms of information handling in neural assemblies involved in computational and cognitive tasks is a challenging problem. Synergetic cooperation of neurons in time domain, through synchronization of firing of multiple spatially distant neurons, has been widely spread as the main paradigm. Complementary, the brain may also employ information coding and processing in spatial dimension. Then, the result of computation depends also on the spatial distribution of long-scale information. The latter bi-dimensional alternative is notably less explored in the literature. Here, we propose and theoretically illustrate a concept of spatiotemporal representation and processing of long-scale information in laminar neural structures. We argue that relevant information may be hidden in self-sustained traveling waves of neuronal activity and then their nonlinear interaction yields efficient wave-processing of spatiotemporal information. Using as a testbed a chain of FitzHugh-Nagumo neurons, we show that the wave-processing can be achieved by incorporating into the single-neuron dynamics an additional voltage-gated membrane current. This local mechanism provides a chain of such neurons with new emergent network properties. In particular, nonlinear waves as a carrier of long-scale information exhibit a variety of functionally different regimes of interaction: from complete or asymmetric annihilation to transparent crossing. Thus neuronal chains can work as computational units performing different operations over spatiotemporal information. Exploiting complexity resonance these composite units can discard stimuli of too high or too low frequencies, while selectively compress those in the natural frequency range. We also show how neuronal chains can contextually interpret raw wave information. The same stimulus can be processed differently or identically according to the context set by a periodic wave train injected at the opposite end of the chain.

## Introduction

Distributed spatiotemporal processing of neural information is widely recognized as the basis for binding and generation of ultimate cognitive abilities in the brain [1], [2]. Gamma waves have been postulated as a carrier of such high order functions [3], [4]. Recently the propagation of solitary waves in two-dimensional neuronal structures has been proposed as a mean for generation of compact internal representations of external dynamic situations [5], [6]. Thus growing evidence suggests that neurons can participate in a collective processing of long-scale information, relevant part of which is shared over all neurons but not concentrated at the single neuron level. In this context we define *wave-processing of information* as a computation (in terms of modification of global information contained in neuronal structure) mediated by nontrivial interaction of waves propagating over neuronal tissue. Thus the brain may actively work not only in time domain but also effectively use spatial dimension for information processing.

Despite wide consensus on significant relevance of long-scale waves for information processing, neurophysiological and biophysical bases of their origin and interaction are largely unknown. Indeed, in the vast majority of experimental and theoretical models, waves traveling over dissipative excitable media (including neuronal structures) vanish at collision (see e.g. [7]–[9]). For example, refractory period behind traveling waves of spreading depression forces their annihilation after collision [10], [11]. Obviously complete destruction of neuronal excitation caused by the interaction of waves cannot contribute to effective and versatile processing of information. A remarkable exception is the backpropagation of action potentials in dendrites involved in plasticity mechanisms and stimulus selection [12]. Recent experimental and modeling results show that annihilation of colliding dendritic spikes, far to be a residual phenomenon, could be crucial for information processing in active dendrites [13], [14]. At the mesoscopic level, recent studies of local field potentials created by synaptic currents in dendrites revealed nontrivial interaction of the confluent inputs to populations of target cells [15]–[17]. Particularly, it has been found that Schaffer input to the CA1 region of the hippocampus is composed of wave trains in the gamma band. Then the coordinated activity of CA3 pyramidal neurons increases information flux in this pathway.

Another handicap for spreading the concept of wave-processing is its scant experimental support due to significant difficulties in detection of macroscopic waves in multi-electrode data and their functional interpretation [18]. Most of the waves described in the literature have pathologic nature and hardly participate in information processing. Examples are large-range epileptic waves, spreading depression of Leao, spiral waves in hart tissue, etc. [11], [19]–[21]. Nevertheless, importance of self-sustained waves propagating and interacting throughout the intricate neuron morphology has been recently put in evidence [4], [22]–[24]. For example, it has been found that sniffing an odor induces three waves at different locations of turtle olfactory bulb [22]. These waves then interact in a complex way. When consecutive odor stimulations are presented, one of the waves is enhanced if the odorants are the same but suppressed if they are different. This finding suggests that waves may carry information about previous olfactory experience and process it appropriately. Thus investigation of mechanisms allowing neuronal structures to represent and process information in a significantly spatiotemporal way is a challenging theoretical and experimental problem with vital impact in different fields of Neuroscience, Medicine, and Nonlinear Dynamics.

One of the most successful approaches for dealing with processing of long scale information uses the FitzHugh-Nagumo (FN) paradigm, which under simple mathematical assumptions captures essential functional features exhibited by neurons. The FN-model has been widely used to describe biological neural networks, interaction and propagation of waves, and processing of information (see e.g. [25]–[27] and references therein). Nonetheless these works assume that neurons locally create information, which is then transmitted, shared, and processed at the network level. We, however, shall demonstrate that nonlinear interaction of self-sustained waves, as carrier of information, can be implemented in classical chains of coupled FN-like neurons. Then such chains modeling laminar neuronal structures acquire ability of wave-processing of long-scale information.

Head-on collision of self-sustained waves in classical FN-chains leads to their complete annihilation. Such monostable interaction offers little, if any, computational capacity, whereas versatile wave-processing of information requires *bistable interaction of waves*. Thus, simultaneously with wave annihilation the network dynamics has to admit at least one more significantly different response to the input stimuli, i.e. traveling waves should be able to cross each other. Transparent crossing of self-sustained waves has been known for a long time. In the last decades it has been shown that such behavior is not exclusive attribute of solitons, but a generic property observed experimentally [28], [29] and numerically [25], [30]–[34]. The mechanism of crossing of self-sustained waves has been attributed to different nonlocal properties of the medium as e.g. cross-diffusion [34].

In this work we show that versatile wave-processing of long-scale information in laminar neural structures, described within the FN-paradigm, can be achieved by introducing into the *single-neuron dynamics* an additional voltage-gated membrane current. This local mechanism, ubiquitous in real neurons [35], provides a chain of such neurons with new emergent network properties. In particular, nonlinear waves as a carrier of long-scale information exhibit a variety of functionally different regimes of interactions from complete or partial annihilation to transparent crossing. Thus neuronal chains can work as computational units performing different operations over spatiotemporal information. To further illustrate the great potential of the concept we show that neuronal chains can “discard” stimuli of too high or too low frequencies, while selectively compress those in the “natural” frequency range, i.e. we observe the phenomenon of *complexity resonance*. We also show how raw wave information can be contextually “interpreted” by a neuronal chain, i.e. the chain can process the same stimulus differently or identically according to the context set by a periodic wave train injected at the opposite end.

### Interaction of Waves in Chains of Coupled Neurons

We shall illustrate the concept of the information wave-processing by using a one-dimensional chain of FN-like neurons:
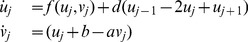
(1)where 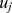 and 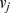 are the so-called membrane potential and recovering variable of the 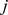
th neuron, respectively; 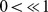 is the smallness parameter; and 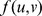 accounts for nonlinear kinetics of the transmembrane currents. Finally 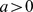, 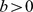; and the parameter 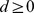 accounts for the strength of couplings between neighboring neurons. The chain (1) is considered with Dirichlet boundary conditions: 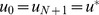, where 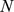 is the total number of neurons in the chain and 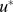 is the resting potential.

### FitzHugh-Nagumo Dynamics

In the original FN-neuron the membrane kinetics is given by:
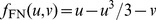
(2)


**Figure 1:**
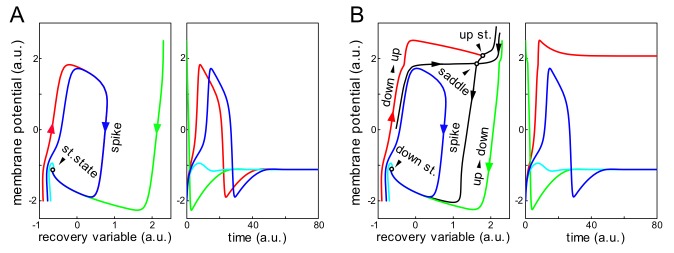
Single neuron dynamics. A) Original FN-neuron. Small but finite excitations can produce spikes (red and blue curves). B) Bistable-excitable neuron (FN-neurons equipped with additional voltage-gated membrane current, 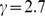). The neuron admits FN-like spikes (blue curve) and transitions between “down” and “up” states (green and red curves).

Setting in (1) 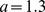 and 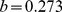 (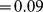, 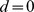) we ensure that single FN-neuron has a unique attractor, a stable steady state, given by
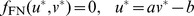
where 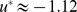 a.u. defines the resting potential. Any perturbation of the neuronal state decays to the steady state, however, small but finite excitation can lead to a large excursion in the phase plane, i.e. to a spike (Fig. 1A).

### Voltage-gated Depolarizing High-threshold Current

Let us now introduce into the neuron’s kinetics an additional voltage-gated high-threshold current, e.g. due to Ca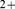 conductance.
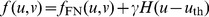
(3)where 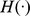 denotes a Heaviside-like step function (we assume 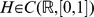), 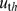 is the voltage threshold (we set 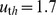 in numerical simulations), and 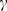 describes the magnitude of the additional current. We note that the extended neuron model with the kinetics (3) reduces to the classical FN-neuron at 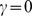.

For 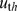 big enough (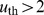 for 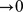) the neuron conserves FN-intrinsic excitable property and can generate spikes similarly to the FN-neuron (Figs. 1A and 1B, blue curves). By rising 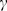 above the critical value:
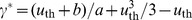
a pair of additional steady states appears on the phase plane of single neuron through a fold bifurcation. Thus the neuron becomes bistable and can stay at rest either in “down” or “up” states, whereas a saddle point separates their basins of attraction. Strong enough perturbations can switch the neuron between down and up states whereas at the down state it can also generate spikes (Fig. 1B). The bistable property of the neuron together with excitability makes the collective dynamics of a chain of such neurons (e.g. interaction of waves) nontrivial.

### Role of Depolarizing Current in Head on Collision of Waves in Neuronal Chains

**Figure 2:**
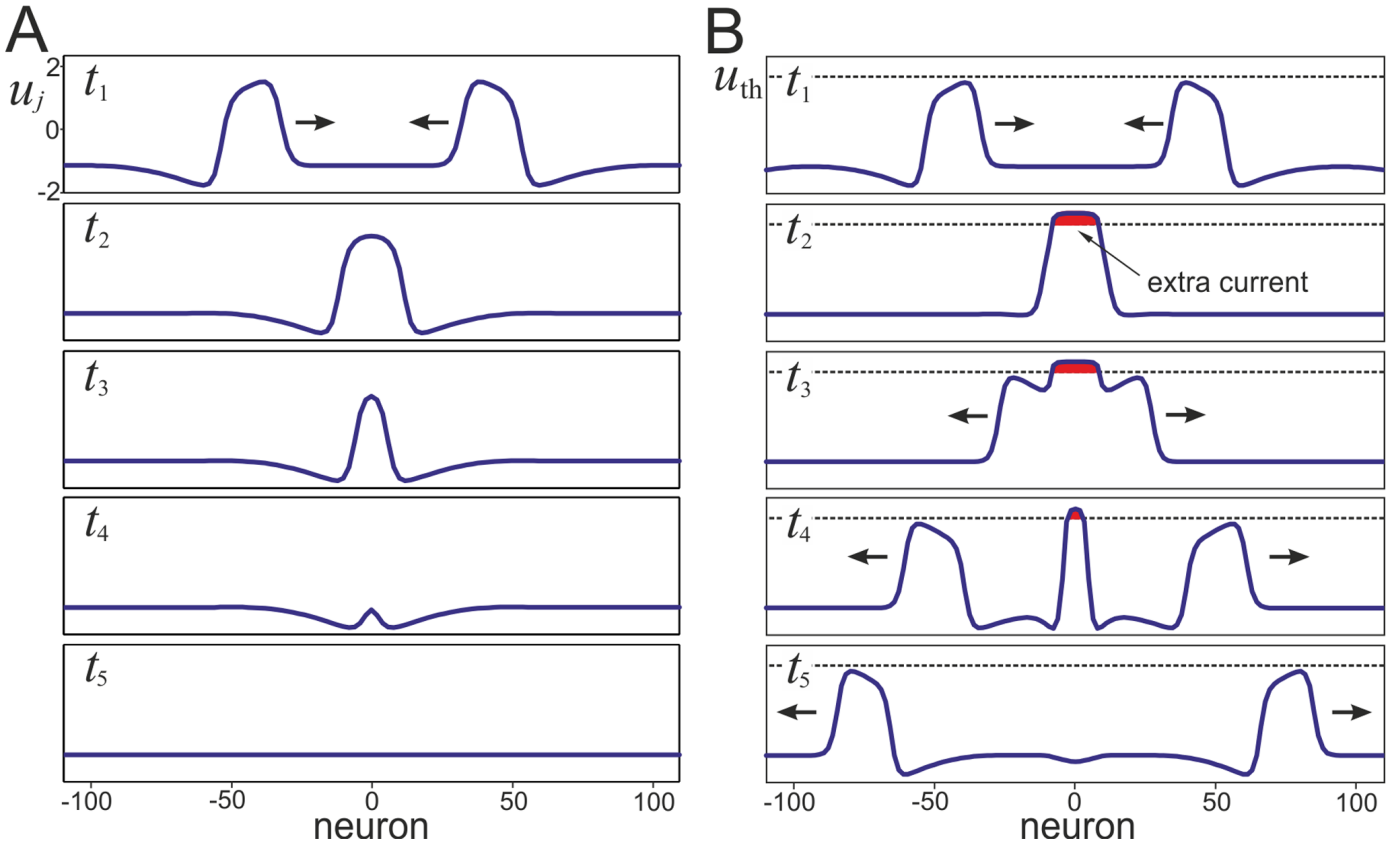
Head on collisions of waves in neuronal chains. A) Snapshots of the membrane potential along the classical FN-chain (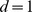) for five consecutive time instants 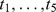. Long scale self-sustained waves travel (arrows mark direction of propagation) and annihilate at collision. B) Wave collision in the chain of bistable-excitable neurons (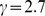, 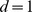). Dashed horizontal line marks the voltage threshold 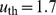. At collision a transient extra membrane current provokes wave regeneration (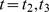). The newly emitted waves propagate in opposite directions, while the wave generator collapses (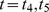).

Classical excitable FN-chain (1), (2) for strong enough coupling, 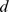, admits self-sustained pulse-like running waves. Figure 2A illustrates head-on collision of such waves, which leads to their annihilation. As mentioned above such behavior is typical for waves with refractory period (see e.g. [8] for general discussion and [10], [19] for electrophisiological and theoretical examples). Thus only trivial wave-processing of information, i.e. its annihilation, can be achieved in this chain.

To cope with this restriction, above we extended the FN-model (1), (3). Figure 2B shows the wave behavior in the chain of bistable-excitable neurons. At the beginning the wave dynamics repeats the classical FN-chain (snapshot 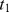). Indeed, in standard conditions of waves propagation the membrane potential 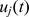 does not reach the threshold 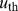 and the extra membrane current in (3) is negligible. Hence no difference exists between the wave behavior of the classical chain and the chain of bistable-excitable neurons. However, when the waves collide, the membrane potential in the collision region overcomes 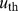 and appearing extra membrane current changes their dynamics (Fig. 2B, snapshot 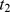).

Balance between the depolarizing membrane current and the axial (along the chain) diffusive current creates a new quasi-stable structure, wave generator (Fig. 2B, snapshot 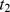). The drive exerted by the wave generator transiently avoids collapsing of the chain excitation and emits two new waves propagating in opposite directions (Fig. 2B, snapshots 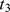, 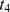). Finally, when the newly created waves run away, the balance between the excitatory and dissipative currents breaks and the wave generator collapses (Fig. 2B, snapshots 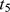).

Thus the relation between the magnitude of the voltage-gated excitatory current controlled by 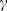 and the axial (coupling) current controlled by 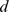 defines the functional regime of the wave collisions. As we shall see below the chain (1), (3) can exhibit a rich repertoire of behaviors and unexpected computational capabilities, which stem from the possibility of waves to cross each other. It is also worth noting that for small enough inter-neuronal coupling 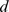 the chain possesses several stationary or quasi-stationary behaviors including variants of spatial chaos (see for details e.g. [36], [37]). We, however, concentrate here on the wave behavior and hence below restrict to the case 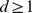.

### Bases of Information Wave-processing

As we shall see further the computational abilities of neuronal chains are based on coexistence of significantly different scenarios of wave collisions. In other words, for effective information processing the chain must admit at least two collision scenarios for the same parameter values. Above (Fig. 2B) we observed one scenario, the wave-crossing, which (in some extent) conserves the information in the chain. Let us now show that the dynamics of the chain of bistable-excitable neurons can be even more complex.

#### Collision scenarios

**Figure 3:**
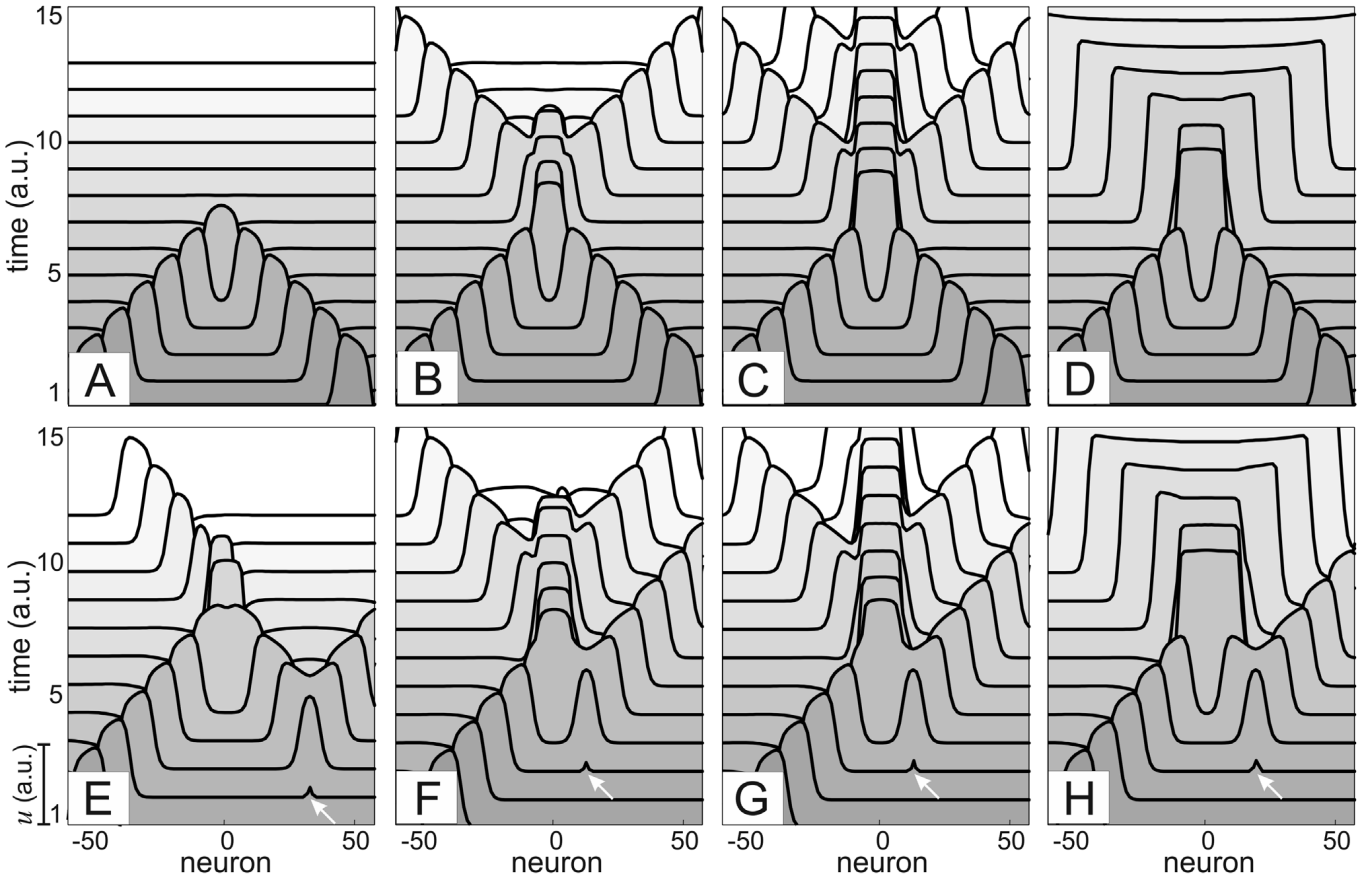
Basic elements of wave-processing of information in the chain of bistable-excitable neurons. A)–D) Scenarios of symmetric head on collision (central part of the chain is shown). The outcome depends on the magnitude of the additional voltage-dependent membrane current: A) Waves annihilate, 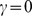 (see also Fig. 2A); B) Waves cross each other, 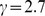 (see also Fig. 2B); C) A pacemaker is formed and emits periodically waves, 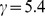; and D) Formation of phase switching supersonic waves, 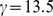. E)–F) Asymmetric collision of a stationary traveling wave with newly created wave (arrows): E) Only one wave survives after collision, 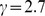; F) Two desynchronized waves emerge in collision, 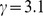; G) and H) the same as (C) and (D) but with desynchronization between waves emitted to the left and to the right (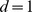).

First, we assume that colliding pulses are stationary waves, i.e. all transient processes of the wave formation have vanished and waves are given by
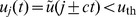
where 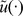 is a pulse-like function and 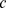 is the wave velocity. Figures 3A–3D show the spatiotemporal evolution of two symmetric colliding waves for different values of the magnitude of additional excitatory membrane current (controlled by 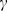).

For small enough 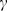 two colliding waves annihilate as it typically happens in the FN-chain in particular and in reaction-diffusion systems in general (Fig. 3A). For moderate values of 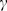 the waves cross each other enabling transparent transmission of wave-information (Fig. 3B). We notice a positive phase-shift at the collision, i.e. delay in the wave reemission. For even higher 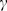 the neurons involved in collision are switched to the up-state and form a pacemaker that emits periodic sequence of waves (Fig. 3C), i.e. a new source of wave-information emerges in the chain at the place of spatial coincidence of waves. Finally for high enough 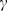 the up-state becomes dominating and two phase waves emerging at the collision switch the chain from down to up-state (Fig. 3D). Such behavior is similar to waves of spreading depression in the hippocampus [19]. We note that the phase transition is “supersonic”, i.e. it propagates faster than subthreshold “sound” waves.

Second, we consider asymmetric collisions of a stationary traveling wave with a wave newly excited by a stimulus applied near the place of future collision. In general, asymmetric collisions lead to asymmetry in the wave creation. For moderate 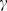 we observe selective annihilation of a part of the information (Fig. 3E *vs* 3B). Such behavior is untypical for solitons and for traveling waves in most of the reaction-diffusion systems (including the classical FN-chain). We also note that the behaviors shown in Figures 3B and 3E correspond to the same parameter values, i.e. the *chain exhibits bistable interaction of waves*, condition required for effective wave-processing of information. For slightly higher 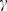 the waves cross each other as in Figure 3B but now the newly created waves are desynchronized, i.e. they receive different phase shifts (Fig. 3F). For the value of 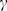 corresponding to the formation of a pacemaker the released waves again have different phase shifts (Fig. 3C *vs* 3G). Similarly, in the phase wave regime the wave emitted to the right has lower phase shift (Fig. 3H *vs* 3D).

#### Bifurcation analysis of wave-processing

The numerically found different collisions’ scenarios (Fig. 3) correspond to functionally different states of the information processing in the chain. In order to gain insight into the dynamics of wave interaction we studied bifurcations occurring in the system.

The stationary solutions of Eqs. (1), (3) are given by the 2D map:
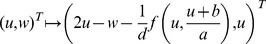
(4)


The map admits three constant solutions (fixed points):
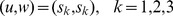
which correspond to the steady states of a single neuron (Fig. 3B), for example, 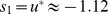 a.u. is the down-state.

**Figure 4:**
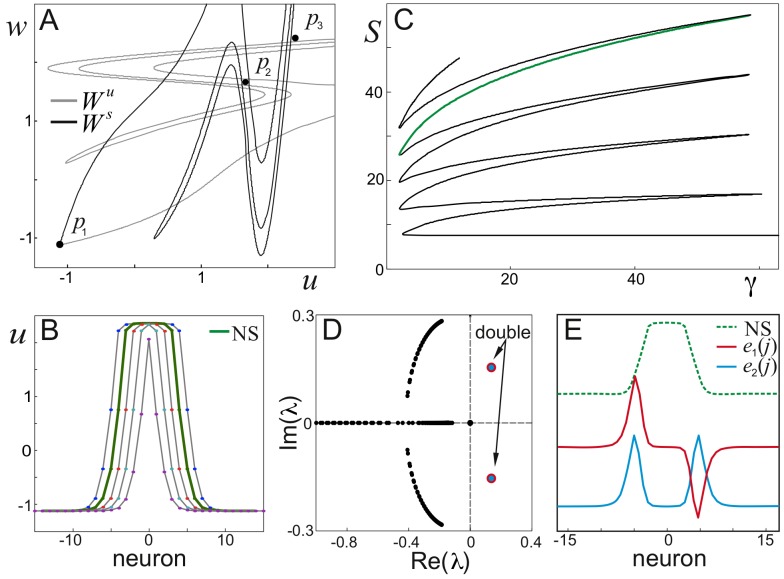
Analysis of the nucleating solution leading to different scenarios of wave collisions. A) Stable and unstable manifolds to the saddle point 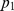 in the map (4). Black dots mark three fixed points (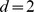, 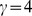). B) Spatial profiles of several homoclinic orbits (NS stands for nucleating solution). C) Bifurcation diagram. Each branch corresponds to a homoclinic orbit in the map (4). D) Eigenvalues of the NS. Two pairs have positive real value. E) NS and real parts of the eigenvectors corresponding to the eigenvalues with positive real part: asymmetric, 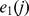, and symmetric, 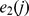.

The fixed point 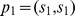 is of a saddle type. There exist variety of orbits homoclinic to 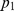. Figure 4A shows stable, 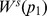, and unstable, 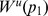, manifolds and their intersections define homoclinic orbits. Several spatial profiles of the homoclinics are shown in Fig. 4B. They differ by the width of the stationary solution and one of them (green in Fig. 4B) corresponds to the width of the wave generator transiently formed during the wave collision (Fig. 4B, 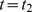). Following Ref. [25] we call such orbit (spatial profile) a nucleating solution (NS).

To describe bifurcations of the homoclinics we introduce the integral characteristics:
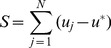
(5)


Then using one of the orbits provided by the intersection of manifolds 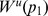 and 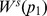 as initial point we continued the homoclinics over the control parameter 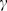 (Fig. 4C). For intermediate values of 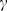 there exist a number of homoclinic orbits, which appear and disappear through fold bifurcations. This analysis shows that there is a critical value of 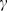 below which there is no nucleation and hence colliding waves annihilate (Fig. 3A).

For nontrivial collisions (Figs. 3B–3H) the existence of an NS is a prerequisite. Under collision trajectory in the phase space of the chain (1), (3) passes nearby the steady state corresponding to NS, which guides the further scenarios of the wave behavior. We then linearized the system (1), (3) in a vicinity of this steady state, which turned to be a saddle. Indeed, its spectrum has one zero-eigenvalue, corresponding to the translation symmetry in the chain, and two pairs of complex eigenvalues with real positive parts (Fig. 4D). Figure 4E shows the corresponding eigenvectors that describe scenarios of the development of instability. Both unstable directions have the same exponent, and hence their winner is determined by how the trajectory enters the saddle region, i.e. by initial perturbation created at the wave collision.

At symmetric collisions (Figs. 3B–3D) the perturbation is also symmetric going along the symmetric eigenvector 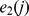 (Fig. 4E). This leads to generation of a pair of symmetric pulses at the tails of the NS. Asymmetric collisions brake the symmetry and the NS will be asymmetrically perturbed, i.e. the initial conditions are shifted to the asymmetric eigenvector 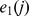. Then we have opposite drive in the tails of the NS, which is the origin of asymmetry in the forming structure. After the first local separation over the unstable manifold, the following behavior of the chain is nonlocal and depends on the controlling parameters.

**Figure 5:**
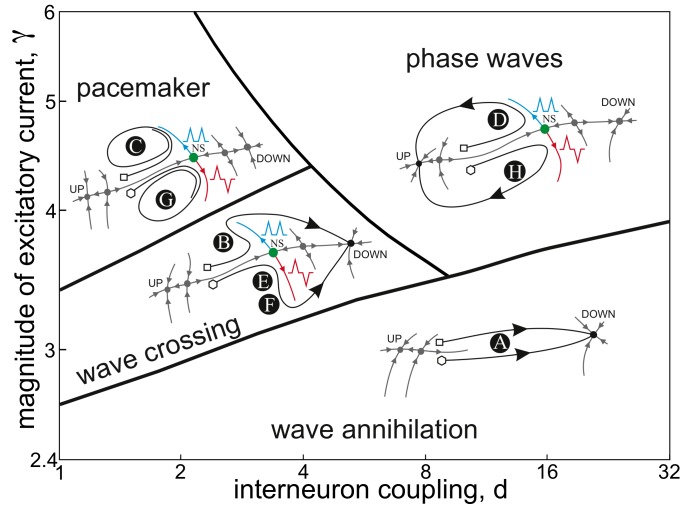
Bifurcation diagram and sketches of trajectories describing different scenarios of wave collisions in the chain of bistable-excitable neurons. In each inset: Square and diamond mark initial conditions for symmetric and asymmetric collisions, respectively; Green dot corresponds to the nucleating solution (NS); Blue and red unstable manifolds represent development of symmetric and asymmetric perturbations (see also Fig. 0E); Letters in black circles correspond to scenarios shown in Fig. 3.

Figure 5 shows complete bifurcation diagram of the neuronal chain (for 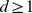). It has four domains with qualitatively different behaviors. In the region of wave annihilation NS does not exist and independently on the collision symmetry the initial perturbations go straight to the down-state, which corresponds to the scenario A in Figure 3. In the remaining domains the NS separates trajectory flows, which gives rise to symmetric and asymmetric scenarios. In the wave crossing domain the unstable manifold of NS pushes the trajectory outside to a big excursion, which results in reemission of two symmetric waves or one single wave or two asymmetric waves (scenarios B, E, and F in Fig. 3, respectively). In the pacemaker domain a limit cycle is born from a saddle-node type bifurcation, which results in emission of periodic waves of finite amplitude (scenarios C and G). Finally in the phase wave domain the trajectories are redirected to the up-state, and hence the chain is switched dynamically to the up-state (scenarios D and H).

Finally we note that one of the most interesting regions, the wave-crossing, extends over quite a big area in the parameter space (Fig. 5). Thus the observed phenomena of the wave-crossing (Figs. 3B and 3E) are robust to variation of e.g. the wave velocity (controlled by 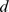) and amplitude.

### Wave-processing of Long-scale Information

As mentioned above, different functional regimes in neuronal chains can be achieved by proper adjustment of the coupling strength between neurons and the membrane voltage-gated current (Fig. 5). One of the most interesting regimes, the wave crossing, occurs for intermediate values of both parameters. In this section we study what computational abilities such functional state may offer.

#### Concurrence of periodic wave trains: Four types of wave-processing

**Figure 6:**
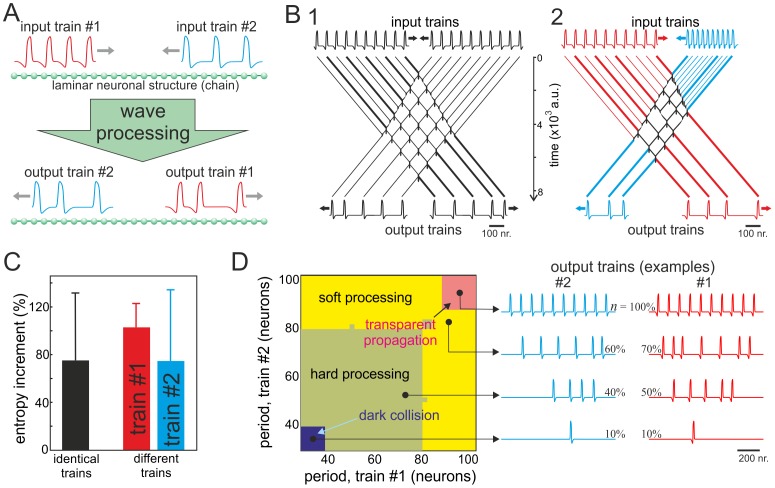
Wave-processing of periodic wave trains: Complexity resonance. A) Sketch of numerical experiments. Two input periodic wave trains (#1 and #2) are injected from both ends of the chain modeling laminar neuronal structure (gray arrows indicate direction of propagation). Nonlinear interaction of the trains, i.e. the wave-processing, leads to two emerging output trains with different aperiodic structure. B) Representative examples of collisions of identical periodic trains (left panel: 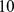 waves, spatial period 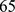 neurons) and trains with different spatial periods (right panel: 10 waves, spatial periods 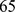 and 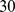 neurons). Evolution of the wave crests shows how the interplay between symmetric and asymmetric wave collisions yields aperiodic output trains. Waves propagating to the output are drawn by thicker lines. C) Entropy increment provided by the wave-processing of identical and different periodic trains (means and standard deviations). For identical trains periods from 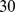 to 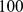 neurons have been considered. For different trains the period of the input train #1 was kept constant (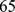 neurons), while the period of the input train #2 spanned interval from 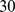 to 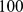 neurons. D) Complexity resonance. Left panel: colored regions mark four types of wave-processing. Right panel: Examples of output trains for each type of the wave-processing. Complexity of the output trains reaches maximum at intermediate spatial periods of input trains (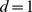, 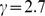).

The real potential of the wave-processing of neural information arises in realistic biological contexts. For example, interaction of coordinated inputs from the lateral and medial entorhinal cortex to the laminar structure of the hippocampus participates in consolidation of memory [38]. Let us now simulate concurrence of two coordinated inputs to a spatially extended laminar neuronal structure. We shall model the information content by two periodic wave trains injected into a chain of bistable-excitable neurons from opposite ends (Fig. 6A). After nonlinear interaction, in general, wave trains change their internal structure and we get two emergent output trains carrying out the processed information.

Figure 6B.1 shows spatiotemporal evolution of two colliding identical periodic trains. Since the chain is in the wave crossing regime (Fig. 5) two collision scenarios are possible: transparent wave crossing with phase shift (Fig. 3B) and annihilation of one of the waves (Fig. 3E). Which of the scenarios is realized in each collision depends on a number of factors, e.g. on the time passed from the previous collision. Indeed, when the spatial period between waves is small enough the newly created waves have no room to stabilize and one of them dies. In contrast, sparse waves (i.e. long time between interactions) cross each other transparently. Thus the proper combination of symmetric and asymmetric crossings is behind the generation of new aperiodic wave patterns at the output. In Figure 6B.1 every odd wave propagates to the output. Thus we can speak about a kind of decimating processing. However, different waves receive different phase shifts in collisions and consequently the structure of the output trains is more complex (aperiodic).

To get deeper insight into the wave-processing we injected into the chain two periodic wave trains as above, but with different inter-wave periods. The train’s asymmetry leads to different dynamic processing of each train and generation of new trains with complex inter-wave structures. Figure 6B.2 shows a representative example of such experiments. Both trains initially had 10 periodic waves spaced by 65 (train #1) and 30 (train #2) neurons. Four waves from the train #1 and three from the train #2 survived at the output. These were number 1, 5, 7, 9 and 1, 7, 10 for the trains #1 and #2, respectively. We also notice significantly different phase shift obtained by each wave, which finally codifies the number of collisions and their frequencies. Thus the neuronal chain can perform nontrivial information processing beyond decimating. It can dynamically select and precisely position in time only “desired” waves from a raw message, which finally convey mutual information in “compressed” form.

In order to quantify the outcome of the wave-processing we introduce an entropic measure. Wave trains at the input and output were converted into binary vectors with ones corresponding to wave crests separated by blocks of zeros (silences). The bin size was equal to the spatial refractory period (20 neurons). Then we evaluated the block entropy [39] over a set of words obtained by sliding a window of 10 symbols over the input and output vectors:
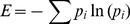
(6)where 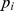 is the relative frequency of the 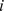
th word. Although this measure for finite trains may underestimate the real train entropy it suits well for our purpose of quantification of the observed information compression. Finally we evaluated the relative variation of the information content before and after wave-processing as:



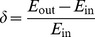
(7)As we expected, during the wave-processing the information contained in wave trains grew significantly (Fig. 6C). The mean growth was about 75% in experiments with identical trains with spatial period varying from 30 to 100 neurons. High variability of the information increment (std 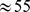) indicates strong dependence of the wave-processing on the inter-wave period.

Collision of wave trains with different periods (Fig. 6B.2) leads to different entropy increments. The train #1, the spatial period of which was kept constant, got 100% mean increment (std 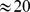), while the train #2, the period of which was changed in the range 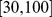 neurons, received 75% increase with std 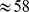. Thus overall characteristics of the wave-processing of the 2*nd* train were similar to the case of identical trains (Fig. 6C). Surprising relatively low variability of the train #1 (std 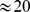) suggests that the informational outcome of the wave-processing of a train depends strongly on its own period but only slightly on the period of the other colliding train. Thus the chain can process information in different spatiotemporal domains, effectively reducing the number of “redundant” waves in one train, while keeping practically constant the informative structure of the other train.

Finally we spanned periods of both colliding trains in the range from 30 to 100 neurons, while keeping 10 waves in each train. As we observed before different number of waves survived after collision. Depending on the proportion of survived waves, denoted by 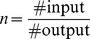, we classified four functionally different types of wave-processing (Fig. 6D):

- *Transparent propagation*. (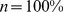, all waves propagate to the output)
- *Soft processing*. (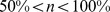, most waves propagate to the output)
- *Hard processing*. (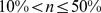, some waves propagate to the output)
- *Dark collision*. (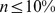, few waves propagate to the output)

For colliding trains with large periods there is room for symmetric wave crossing and no annihilation occurs. Then the output trains are identical to the input ones, i.e. trains transparently cross each other receiving global phase shift (Fig. 6D, red area: transparent propagation). For shorter spatial periods some asymmetric wave crossings appear, which decreases the number of waves propagating to the output (Fig. 6D, yellow area: soft processing). In soft processing at least one train conserves most of the input waves. For intermediate periods of both input trains the wave-processing, denominated as *hard processing* (Fig. 6D, green area), leads to annihilation of the majority of input waves. Finally for really short periods (Fig. 6D, blue area: dark collision) annihilation dominates the wave-processing and only few (usually only the first) waves propagate to the output.

Transparent propagation does not alter the complexity measure (6) and hence 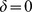. Soft and hard processing regimes increase significantly the informational content at the output, i.e. 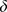 is high, whereas dark collision leads again to 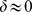. Thus we have a kind of band-pass filtering of periodic waves, but instead of simple reduction of the train period we have changes in the train complexity. For intermediate spatial periods the information is maximal and then decreases for long and short periods. Such *complexity resonance* is reminiscent of the rate-temporal coding problem (see e.g. [40]). Indeed, our neuronal structure can “ignore” stimuli of too low frequency and “annihilate” those of too high frequency, while selectively process stimuli in the “natural” frequency range. The processed stimuli are compressed and get higher train complexity at the output.

#### Context dependent information processing

A remarkable quality of evolved living beings is their ability to *interpret* information according to circumstances. Response of an organism to the same stimulus can depend on, for example, its internal state or external situation. Then the context acts like a framework for such high-level functions as learning, memory, understanding, etc. [6], [41]. The proposed concept of wave-processing of information also includes contextualization as one of its central features.

**Figure 7:**
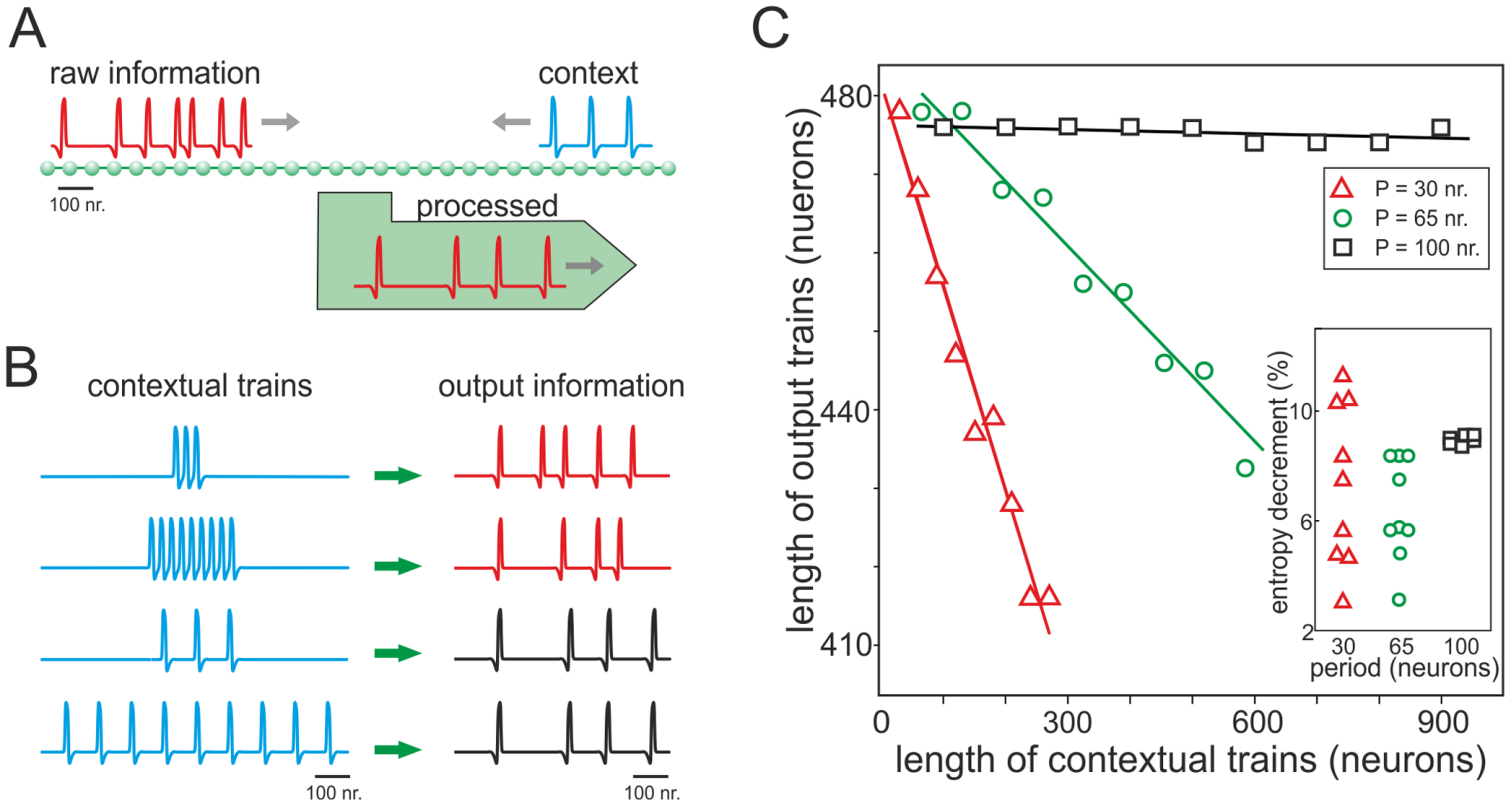
Contextual processing of information. A) Sketch of numerical experiments. The wave train injected at the left input of the neuronal chain conveys raw information (stimulus to be processed, the same for all experiments). The informative train interacts with a periodic train injected at the right input, which sets the context for the wave-processing. The context features are the spatial period and length of the contextual train. After trains’ interaction the processed information can be readout from the right end of the chain. B) Four examples of different contexts (trains with 3 and 9 waves spaced by 30 and 100 neurons) and the corresponding outputs (processed trains). Red trains are different, whereas black train are the same, which suggests divergence/convergence of the contextualization. C) Influence of the context on the information extracted from the raw message. Main graph: the length of the output train linearly depends on the length of the contextual train and its period modulates the sensitivity. Inset: relative entropy of the output has higher variability for contextual train with shorter periods (horizontal displacements of symbols serve for visualization only).

To illustrate how contextualization of raw long-scale information can be implemented in neuronal structure, we used again the two-inputs paradigm (Fig. 7A). Left end of the neuronal chain has been designated as an informative input, i.e. it receives information or stimulus to be processed by the chain. The purpose of the right end is dual. It is used: i) as an input for contextual trains and ii) for readout of the computation results. While the informative train can have rather complex aperiodic structure and consequently high entropy, the contextual train may be quit simple. In all experiments we employed the same informative train shown in Figure 7A (raw information), whereas for setting different contexts we used periodic wave trains with different number of waves and inter-wave periods (Fig. 7B, left trains).

In general, interaction of the informative train with different contextual trains leads to different output trains (Fig. 7B, red trains). The output trains convey information coded in the raw stimulus but modulated by the context. Thus the output message is a contextualized variant of the input information. Although different contexts usually yield different outputs, we notice that the same output may also occur (Fig. 7B, black trains). Such simultaneous divergence/convergence of the contextual information processing is also known in the Nature. Indeed, organisms may act differently or identically to the same stimulus in different circumstances.

In order to illustrate the great potential of the contextual wave-processing of information we performed the following experiments. Using the same input stimulus with high entropy (Fig. 7A, raw information) we tested contextual trains of different spatial length with three different spatial periods: 30, 65, and 100 neurons. To quantify changes in the output wave train we employed two measures: i) the spatial length (related to compression) and ii) the relative entropy (related to complexity). Figure 7C summarizes our results.

We found that the length of the output train changes practically linearly with increase of the length of the contextual train. The slope of the least-squares linear regression strongly depends on the period of the contextual train. Contextual trains with the shortest spatial period of 30 neurons (Fig. 7C, red triangles) exert strongest impact on the length of the output (processed) train, whereas trains with longest spatial period of 100 neurons have little effect on the output train (Fig. 7C, black squares). To confirm this observation we also evaluated the relative entropy (7). Since the input stimulus (raw information) has high entropy, in this case the wave-processing led to entropy decrease (Fig. 7C, inset), i.e. the wave-processing selects only a part of input information. In agreement with previous results, we observed that the variability of the output information is maximal for contextual trains with short period (30 neurons) and minimal for trains with long period (100 neurons). Thus the neuronal chain offers effective mechanism for contextualization of the input information. We can easily control characteristics of the processing by changing the length of the contextual wave-train and tune the sensitivity to the context by changing its period.

## Discussion

Questions “How information is represented in the brain?” and “What are the principles of its processing?” are the most challenging in contemporary Neuroscience. It is now well accepted that different brain nuclei use different strategies for information handling. At the initial processing levels, primary brain nuclei codify sensory information in the form of spike trains. At this stage variants of the rate and time coding schemes are largely employed (see e.g. [40], [42], [43] and references therein). However, at upper levels the situation becomes much more complicated. Highly evolved nuclei involve distributed parallel processing of multimodal and multiscale information. Complex networks made up of proximal and distant heterogenous couplings coordinate neural activity at different sites [44]. Then the synchronization concept standing on correlated firing of multiple spatially distant neurons (see e.g. [3]) has been widely spread as a paradigm for computational and cognitive tasks. Although this hypothesis received strong experimental and theoretical support, not all experimental facts can be easily fitted in the paradigm.

It seems that besides synergetic cooperation of neurons in time domain, e.g. through synchronization of spikes in different time windows, the brain may also employ information coding and processing in spatial dimension. For example waves of neural activity, functionally related to behaviors and global dynamics, have been found in visual, sensory-motor, auditory, and olfactory cortices (see [24] for a review). In this work we proposed and theoretically illustrated a novel concept of significantly spatiotemporal representation and processing of long-scale information in laminar neuronal structures. We argued that relevant long-scale information may be hidden in spatiotemporal waves, abundant in different brain structures, and then nonlinear interaction of such waves yields efficient information processing, which we called wave-processing. We note that the discussed wave-processing cannot be reduced to the synchronization paradigm since it occurs in two dimensions: space and time, i.e. the result of computation depends significantly on the spatial distribution of information.

To implement wave-processing in a mathematical model we proposed a mechanism that relays on local single neuron dynamics. We incorporated into the classical FitzHugh-Nagumo neuron an additional membrane current accounting for the dynamics of voltage gated high threshold ionic channels. Then a chain of such neurons acquires new emergent properties. Namely, we have shown that nonlinear self-sustained waves can exhibit a variety of functionally different regimes of interactions from complete or partial annihilation to transparent crossing. We provided a rigorous description of the bifurcations in the phase space of the corresponding dynamical system leading to different collision scenarios. It is worth noting that the model incorporates two types of multistability: of a single neuron (Fig. 1B) and of the wave collision (Figs. 3B, 3E). The existence of the former is not essential, i.e. the main results can be reproduced with a monostable single-neuron model (without up state). However, the additional high-threshold conductance is a must for the multistable wave interaction. We have shown that the latter multistability, as basic computational requisite at the network level, is governed by a special nucleating solution of a saddle type with two generic routs leading to different scenarios of wave interaction. Thus besides symmetric transparent wave crossing the neuronal chain simultaneously admits asymmetric wave interaction, an asset for wave-processing. This regime of wave interaction occurs for intermediate (biologically plausible) values of the coupling strength between neurons and the amount of the additional membrane current.

We have shown that neuronal chains can exhibit nontrivial computational abilities mimicking different physiological processes in the brain. In particular we described the phenomenon of complexity resonance and classified four available types of processing of wave information: Transparent propagation, Soft and Hard processing, and Dark collision. Using these “computational tools” a laminar neuronal structure can “ignore” stimuli of too high or too low frequencies (or spatial scales), while selectively process those in the “natural” frequency range. Input stimuli are compressed and receive higher complexity at the output thus effectively codifying raw information.

We have also shown that the concept of wave-processing naturally offers an effective mechanism for contextual computations, i.e for interpretation of raw information according to circumstances or context that acts like a framework for high-level functions. We illustrated contextualization of raw long-scale information using a complex stimulus as input information and periodic wave trains modeling different contexts. We have shown that the content of the output wave train linearly depends on the length of the contextual train and the sensitivity to the context is controlled by the context frequency. As it happens in the Nature contextualization of information obeys divergence/convergence properties. The neuronal chain can process stimulus differently or identically in different circumstances.

Thus neuronal chains can work as computational units performing different operations over spatiotemporal information. Both the biophysical basis of the model and its revealed computational features make it suitable for functional description of global and sparse information processing in real neural networks. We expect that the concept of wave-processing could be involved in such high-level brain functions as path-planning and decision making. Indeed, to behave efficiently and actively in complex environments, evolved organisms create in the brain a model of the external world. Then this model is used to perform mental “computations” and test in parallel different decision alternatives (see e.g. [6] and references therein). To perform this task the brain should be able to map 4-dimensional space-time structure of the external world into the internal neuronal space. Then it seems reasonable to hypothesize that laminar brain structures (like e.g. cerebral cortex) may naturally serve as a container for the information mapping, while neural waves may perform parallel computations over such space-time information.
